# Characterization of *Pseudomonas aeruginosa* Isolated from Bovine Mastitis in Northern Jiangsu Province and Correlation to Drug Resistance and Biofilm Formability

**DOI:** 10.3390/ani14223290

**Published:** 2024-11-15

**Authors:** Yicai Huang, Pengqiang Chen, Hainan Cao, Zheng Zhou, Tianle Xu

**Affiliations:** 1 Joint International Research Laboratory of Agriculture and Agri-Product Safety of Ministry of Education of China, Yangzhou University, Yangzhou 225009, China; 2 College of Animal Science and Technology, Yangzhou University, Yangzhou 225009, China; 3 Fujian Nanxing Animal Health Products Co., Ltd., Nanping 353000, China; 4 Department of Animal Science, Michigan State University, East Lansing, MI 48824, USA; 5 International Joint Research Laboratory in Universities of Jiangsu Province of China for Domestic Animal Germplasm Resources and Genetic Improvement, Yangzhou 225009, China

**Keywords:** dairy mastitis, *Pseudomonas aeruginosa*, MLST, biofilm, virulence genes

## Abstract

This study aimed to provide experimental support for the prevention and treatment of *Pseudomonas aeruginosa* infections and to elucidate the epidemiological distribution of resistance and virulence genes of *Pseudomonas aeruginosa* from mastitis in dairy cows in the northern part of Jiangsu Province and their relationship with the biofilm-forming ability of the strains. Mastitis presents a significant challenge within dairy farming, adversely impacting the health of dairy cows and precipitating substantial economic losses in milk production. In this study, *Pseudomonas aeruginosa* (*PA*) was isolated and identified from mastitis milk samples in Jiangsu Province, China. In order to characterize the isolates, multilocus sequence typing (MLST), drug resistance phenotypes, virulence genes, and biofilm formations were detected. The isolation and identification of pathogenic bacteria from 168 clinical mastitis milk samples using 16S rRNA and PCR revealed 63 strains of *Pseudomonas aeruginosa*, which were determined to be highly homologous according to phylogenetic tree analysis. In addition, the MLST indicated five major ST types, namely ST277, ST450, ST571, ST641, and ST463. The susceptibility to 10 antimicrobials was determined, and it was found that 63 strains of *Pseudomonas aeruginosa* did not have a strong resistance to the antimicrobials in general. However, there were differences in the phenotypes’ resistance to antimicrobials among the different ST types. It was also found that the more resistant the strains were to antimicrobials, the lower the carriage of virulence genes detected. The biofilm content was measured using the semi-quantitative crystal violet method. It was found that there were a few strains with medium or strong biofilm-forming abilities. However, the number of virulence genes carried by the 63 strains of *Pseudomonas aeruginosa* was inversely proportional to the biofilm-forming ability. It was also found that there were significantly more *Pseudomonas aeruginosa* in the biofilm state than in the planktonic state and that strains with strong biofilm-forming abilities were more resistant to antimicrobials.

## Simple Summary

Mastitis is one of the most prevalent diseases, causing huge economic losses in the dairy farming industry. Various pathogens can cause mastitis in dairy cows, which can be classified as contagious and environmental. The current study used the contagious pathogen *P. aeruginosa* isolated from the milk of dairy cows with clinical mastitis from Jiangsu Province to investigate virulence gene expression and test antimicrobial susceptibility. The potential links among virulence gene expression, resistance of antibiotics and biofilm formability were also uncovered phenotypically or genotypically. The study provides insight into the spread of infection by *P. aeruginosa* and resistance in the case of bovine mastitis, as well as the potential target genes involved in the links between resistance determinants and multi-drug resistance.

## 1. Introduction

Bovine mastitis is one of the most common syndromes affecting milk yield and quality. It can be mainly classified into clinical mastitis and subclinical mastitis according to the classification of the National Mastitis Council [1]. Its clinical manifestations include milk content alteration, reduced lactation, and tissue damage [2]. This disease is closely related to farm management and the abundance of environmental microbes. Its irreversible damage to udders of dairy cows has severely hampered the progress of the dairy industry, with more than one-third of the world’s dairy cows being affected to varying degrees by mastitis, leading to significant global economic losses annually [3]. Antibiotic therapy is used to treat mastitis on most farms, and the accurate detection of mastitis pathogens for symptomatic treatment is regarded as the most efficient approach. The severity of bovine mastitis is usually determined by the species of isolates that are derived from the infected udders. Due to the mixed infections in most cases, opportunistic pathogens such as *Pseudomonas aeruginosa* have raised significant concerns.

*Pseudomonas aeruginosa* is a Gram-negative bacterium that is widespread in a variety of environments and is adapted to harsh conditions [4]. As an opportunistic pathogen, it can cause infections such as mastitis in dairy cows when immune function is compromised [5,6]. For *Pseudomonas aeruginosa*, the use of its physiological and biochemical properties and culture characteristics, as well as modern molecular typing techniques such as multilocus sequence typing (MLST) [7], can help to identify bacterial origins and transmission routes and even optimize prevention and treatment strategies. MLST characterizes microbial isolates by sequencing internal fragments of housekeeping genes [8], revealing significant genetic differences between strains. The resistance rate and antimicrobial spectrum of *Pseudomonas aeruginosa* are enhanced with the long-term use and overuse of antimicrobials, subsequently leading to the development of bacterial resistance mechanisms [9]. Its pathogenic mechanisms are diverse, including surface virulence factors (e.g., flagella, hyphae, lipopolysaccharides, alginate, membrane proteins [10]) and secreted toxins (e.g., alkaline protease secreted by T1SS of the type I secretion system [11], exotoxin A, and elastase enzyme; phospholipase C secreted by T2SS of the type II secretion system [12]; and the effector proteins ExoS secreted by T3SS of the type III secretion system, ExoT, ExoU, ExoY, and pyocyanin [13]), and the form of infection they cause varies according to the type of toxins secreted. However, the relationship between the pathogenic virulence and the carriage of gene-encoded toxins in bovine-mastitis-derived *Pseudomonas aeruginosa* remains uncovered.

*Pseudomonas aeruginosa* is one of the dominant biofilm-forming bacteria [14]. The biofilm is composed of microcolonies immersed in an extracellular matrix, which plays a crucial role in providing structural integrity and resistance to external stresses. This extracellular matrix not only protects the bacterial communities from environmental threats but also facilitates the transport of nutrients and removal of metabolic waste [15]. Biofilm formation significantly enhances bacterial resistance to antimicrobials and helps evade host immune responses, making infections more persistent and difficult to eradicate [16]. In this study, the epidemiological survey of bovine mastitis was conducted on farms in northern Jiangsu Province to reveal the epidemiological trends and characteristics of isolated *Pseudomonas aeruginosa*, as well as the epidemiological distribution of drug resistance and virulence genes, and their relationship with the biofilm-forming ability of the strain.

By integrating MLST with an in-depth analysis of antimicrobial resistance, biofilm formation, and virulence gene profiles, this work provides a comprehensive assessment of bovine mastitis-derived *Pseudomonas aeruginosa*. The dual approaches reveal significant correlations between biofilm formation and drug resistance, as well as the relationships that have been insufficiently explored in previous studies. These findings enhance the understanding of *Pseudomonas aeruginosa* pathogenicity in bovine mastitis and the complex interplay between biofilm formation and resistance mechanisms, suggesting potential strategies for improved treatment and prevention of mastitis in dairy cows.

## 2. Materials and Methods

### 2.1. Bacterial Isolation and Identification

Holstein dairy cows with udder erythema, pathological milk changes, and positive CMT mastitis test were selected from four farms in northern Jiangsu Province. The first few streams of milk from each teat were discarded before sampling, and the collected milk samples were transported to the laboratory for testing within 24 h. Milk samples were collected in 80 μL quantities, spread evenly on blood agar medium using a swab, and incubated in an inverted incubator at 37 °C for 16 h. Obvious colony characteristics including shape, wetness, dryness, and haemolysis were recorded. The single colony was inoculated into a nutrient broth medium by picking with the sterile tip of a pistol, placed in a shaker at 37 °C and 180 rpm, and incubated with shaking for 18 h. The turbid bacterial solution was dipped into an inoculation ring and spread onto Luria–Bertani agar medium and incubated in an inverted 37 °C incubator for 16 h. The purified bacterial solution was obtained by picking a single colony into the Luria–Bertani (LB) broth and incubating it for 18 h with shaking. Genomic DNA was extracted from the isolated *Pseudomonas aeruginosa* strains using the bacterial genomic DNA extraction kit (DP302-02) from TIANGEN Co., Ltd. (Beijing, China), according to the manufacturer’s protocol. Briefly, overnight bacterial cultures were harvested by centrifugation, and the resulting cell pellets were resuspended in buffer and treated with lysozyme for cell lysis. Proteinase K was then added to digest proteins, and the DNA was purified using spin columns. The purified DNA was eluted in elution buffer and stored at −20 °C for subsequent use in PCR and sequencing. The extracted bacterial DNA was used as a template for PCR amplification. The primers for 16S rDNA determination are shown in Table S1, and the PCR amplification system and conditions were as follows: The total reaction volume was 50 μL, which included 22.5 μL of 2× Taq Master Mix, 2 μL of upstream primer, 2 μL of downstream primer, 1 μL of DNA template, and 22.5 μL of ultra-pure water. The PCR amplification program consisted of an initial denaturation at 98 °C for 2 min, followed by 30 cycles of denaturation at 98 °C for 10 s, annealing at 58 °C for 30 s, and an extension at 72 °C for 40 s. A final extension was performed at 72 °C for 1 min. The PCR products were verified by 1% agarose gel electrophoresis and sequenced by Nanjing Tsingke Biotechnology Co., Ltd. (Nanjing, China). The sequences were compared by BLAST on the NCBI database (https://www.ncbi.nlm.nih.gov/) (accessed on 22 March 2023) to determine the genus of the pathogenic bacteria. The forward and reverse sequences obtained by amplification and sequencing of the 16S rDNA gene of *Pseudomonas aeruginosa* were spliced by Contig Express 9.1 software, and then the spliced sequences were compared by MEGA 7 software, and finally the evolutionary tree was constructed in MEGA (Kumar, Stecher, and Tamura, 2016) [17].

### 2.2. MLST Typing

The genomic DNA extracted from *Pseudomonas aeruginosa* was used as the template for PCR amplification, and seven housekeeping genes of *Pseudomonas aeruginosa* for MLST analysis were selected: *acsA*, *aroE*, *guaA*, *mutL*, *nuoD*, *ppsA*, and *trpE* [18]. The primer sequence is shown in Table S2. The amplification conditions were as follows: 96 °C pre-denaturation for 1 min; then 30 cycles of denaturation at 96 °C for 1 min, annealing at 55 °C for 1 min, and an extension at 72 °C for 1 min; and a final extension at 72 °C for 10 min. The PCR products were sent to Nanjing Tsingke Company for sequencing. After sequencing, the validated sequences of the PCR products were submitted to the MLST database (https://pubmlst.org/) (accessed on 2 May 2023) of *Pseudomonas aeruginosa* to obtain the allele number of each housekeeping gene. The allele number of each *Pseudomonas aeruginosa* housekeeping gene was followed in the order of acsA-aroE-guaA-mutL-nuoD-ppsA-trpE to form an allelic profile, which was compared with the existing allelic profiles in the database to obtain the sequence type of the strain following methods described in studies [19]. The phylogenetic tree of the 63 *Pseudomonas aeruginosa* strains was constructed using the neighbor-joining (NJ) method. To assess the reliability of the tree, the bootstrap method was applied with 1000 replicates. Bootstrap values were calculated to provide confidence estimates for each branch in the tree.

### 2.3. Phenotypic Determination of Drug Resistance

The antimicrobials susceptibility of *Pseudomonas aeruginosa* was determined using the K-B paper diffusion method. Antimicrobial-impregnated paper was placed on Mueller–Hinton agar medium inoculated with *Pseudomonas aeruginosa*, and inhibition zones were measured after incubation. All strains were classified as susceptible (S), intermediate (I), and resistant (R) according to the Clinical and Laboratory Standards Institute (CLSI) interpretation criteria [20]. The drugs tested were streptomycin, imipenem, meropenem, gentamicin, piperacillin, penicillin (all 10 µg/tablet), tetracycline, ceftazidime, ceftazidime, ceftazidime/boric acid, ceftazidime/boric acid (all 30 µg/tablet) and ciprofloxacin (5 µg/tablet), supplied by Hangzhou Microbiology Reagent Co., Ltd. (Hangzhou, China).

The selection of these antibiotics was based on their widespread use in veterinary medicine, particularly in treating bovine mastitis, and their efficacy against *Pseudomonas aeruginosa*. Additionally, combinations of antibiotics were tested based on known resistance mechanisms in *Pseudomonas aeruginosa*, including the use of beta-lactamase inhibitors (e.g., ceftazidime/boric acid) and drugs from different classes to target multiple resistance pathways. The antibiotics were selected to represent a broad spectrum of commonly used antimicrobials, including aminoglycosides, carbapenems, and fluoroquinolones, which are critical in both human and veterinary medicine.

### 2.4. Investigation of Virulence Genes in Isolated Pseudomonas Aeruginosa

The genomic DNA of *Pseudomonas aeruginosa* was used as a template, and 15 virulence genes, including the major toxin genes of *Pseudomonas aeruginosa*, such as exoS, exoT, exoU, exoY, Pyo, aprA, toxA, lasA, lasB, plcH, algD, and the regulatory genes of the quorum sensing system (lasI, lasR, rhlL, rhlR), were selected for PCR amplification. The sequence information of the primers is shown in Table S2. The amplification conditions were as follows: 94 °C pre-denaturation for 5 min; then 35 cycles of denaturation at 94 °C for 40 s, annealing at different temperatures for 45 s, and an extension at 72 °C for 2 min; and a final extension at 72 °C for 10 min. The PCR products were validated by 1% agarose gel electrophoresis and sent to Nanjing Tsingke Company for sequencing. These genes were selected based on their well-documented role in the virulence mechanisms of *Pseudomonas aeruginosa* during infection. Specifically, exoU, toxA, and lasR have been frequently linked to severe infections and high levels of antimicrobial resistance. Genes involved in biofilm formation, such as lasI and rhlR, were included to investigate their potential correlation with antibiotic resistance in *Pseudomonas aeruginosa* strains isolated from mastitis.

### 2.5. Biofilm and Cell Viability Assay

In this experiment, the microtiter plate method was used to cultivate biofilm. *Pseudomonas aeruginosa* was inoculated into 24-well plates containing LB broth to form a complete biofilm, and the content of the biofilm was measured using the semiquantitative crystal violet method [21]. Enzymatic markers were used to stain the biofilm matrix and bacterial cells, which allows for the visualization and quantification of biofilm formation. The ability of the strain to form biofilm was identified according to the magnitude of the absorbance value. First, a solution of *Pseudomonas aeruginosa* was cultured overnight, adjusting the OD600 to 0.1 using broth, which corresponds to 1 × 10^8^ cells/mL. Then, 1 mL of this bacterial suspension was transferred into a 24-well plate, creating three replicate wells per bacterial strain, alongside a negative control group containing only broth, and incubated at a constant temperature of 37 °C for 48 h. Upon culture completion, the supernatant was aspirated from each well, followed by triple rinses with 1.5 mL of phosphate-buffered saline (PBS) per well and an air-dry for 15 min at ambient temperature. A total of 2.5 mL of a 75% methanol solution was introduced for fixation over 15 min; post methanol absorption, it was allowed to air-dry for another 15 min at ambient temperature. Subsequently, 3 mL of a 1% crystal violet staining solution (Beyotime, Shanghai, China) was added, staining for 5 min. After staining, the solution was discarded, and the wells were rinsed three times with sterilized ultrapure water before being air-dried at 37 °C for 5 min. Subsequently, 1 mL of 30% acetic acid was added, and the plate was agitated until the stain was completely dissolved. Absorbance at 590 nm was measured for each well using a microplate reader. The optical density (OD) for each strain was calculated as the average of three replicates. The critical value (OD_C_) was defined as the mean OD of the negative control plus three standard deviations (SD) (OD_C_ = OD₀ + 3SD). The relative index (SI) was determined as the ratio of OD to OD_C_ and categorized into three levels [22]:

(1) If 0 < SI ≤ 1.5, it represents a weak or no biofilm formation ability.
(2) If 1.5 < SI < 2.5, it represents a moderate biofilm formation ability.
(3) If SI ≥ 2.5, it represents a strong biofilm formation ability.

### 2.6. Quality Control Procedures

To ensure accuracy and reproducibility, the following control measures were implemented throughout the study: For bacterial isolation, 16S rDNA amplification, virulence gene detection, and negative controls (no DNA) were included to check for contamination, and positive controls confirmed reliable amplification and sequencing. For antimicrobial susceptibility testing, *Pseudomonas aeruginosa* ATCC 27853 was used to validate antibiotic performance and K-B paper diffusion consistency. All culture media were tested for sterility, and PCR reagents were stored properly and checked for expiration. Blank media plates were incubated to ensure sterility under experimental conditions. All procedures were performed in a Class II biological safety cabinet sterilized with UV light and cleaned with 70% ethanol. Instruments were autoclaved, and samples were processed within 24 h to maintain bacterial viability. Sterile 24-well plates were used, with triplicate wells per strain. Negative control wells with only broth confirmed the absence of contamination. PCR products were sequenced and validated by Nanjing Tsingke Biotechnology Co., Ltd. (Nanjing, China), an ISO 17025-accredited reference laboratory for molecular testing [23]. High-quality sequencing and verification were ensured through this accreditation. These sequencing results were vali-dated by comparing forward and reverse sequences using BLAST.

## 3. Statistical Analysis

To explore the relationship between antibiotic resistance phenotypes, biofilm formation abilities, and the number of virulence genes, we conducted a statistical analysis using the Chi-square test. This analysis was performed in SPSS version 26 by selecting the “Crosstabs” option from the “Descriptive Statistics” menu. The Chi-square test helped us calculate the Chi-square value (χ^2^) and the corresponding *p*-value to assess the independence of these variables. We considered a *p*-value of less than 0.05 as statistically significant, indicating a meaningful association between the variables.

## 4. Results

*Pseudomonas aeruginosa* isolates from milk-derived samples were identified, and MLST was performed.

**Figure 1:**
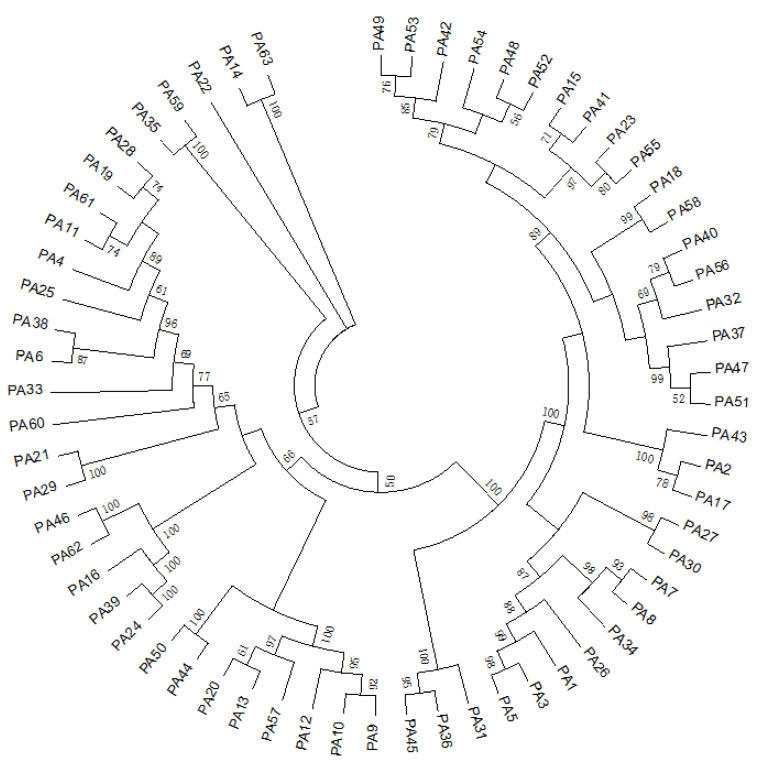
The evolutionary tree of 63 *Pseudomonas aeruginosa.* The phylogenetic tree was constructed using the neighbor-joining (NJ) method. The bootstrap method was used for 1000 tests and the number on the branch is the Bootstrap value.

In this study, 168 clinical mastitis milk samples collected in northern Jiangsu Province were used to isolate pathogenic bacteria using 16S rDNA identification. The results showed that a total of 424 bacterial strains were isolated, among which 63 strains of *Pseudomonas aeruginosa* were detected, with a detection rate of 14.86% (Table S3). Based on the amplified 16S rDNA sequences, a phylogenetic tree of the 63 *Pseudomonas aeruginosa* strains was constructed. As shown in Figure 1, the 63 *Pseudomonas aeruginosa* strains clustered into a few branches, indicating a high degree of genetic homogeneity. Most strains were grouped closely together, suggesting limited genetic diversity among the isolates.

The results of the MLST (Table S4) showed that 63 strains of *Pseudomonas aeruginosa* belonged to 16 ST types. There were five ST types with more than five strains, including ST277, ST450, ST571, ST641, and ST463. Among these major ST types, the largest number of strains was found in ST277. Six ST types, namely ST256, ST594, ST640, ST752, ST838, and ST2197, were represented by only one strain each.

### 4.1. Correlation of Antimicrobial Susceptibility and ST Type of 63 Pseudomonas aeruginosa Strains

The results of the antimicrobial susceptibility test (Table 1) showed significant differences in the resistance rates of the 63 *Pseudomonas aeruginosa* strains to the 10 antimicrobials tested. Among them, the highest resistance rate was found for penicillin, with all strains (100%) showing resistance. Moreover, the resistance rates for streptomycin and gentamicin were 69.84% and 49.21%, respectively. In contrast, the resistance rates of *Pseudomonas aeruginosa* to ciprofloxacin, imipenem, meropenem, and ceftazidime were all 0%, indicating complete sensitivity to these antimicrobials. Meropenem and ceftazidime demonstrated the most pronounced inhibitory effects on *Pseudomonas aeruginosa* growth. Additionally, a relatively high number of strains showed moderate sensitivity to ciprofloxacin, gentamicin, and piperacillin, accounting for 10–20% of the total. Only seven strains (11.11%) were identified as being extended-spectrum beta-lactamase (ESBL) producers, determined by using the disk diffusion method combined with CLSI guidelines for human isolates.

**Figure 2:**
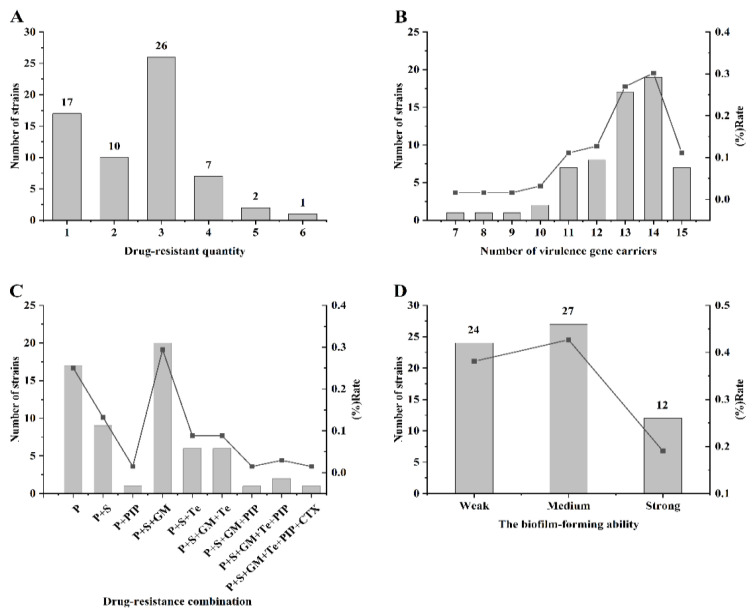
Comprehensive analysis of 63 *Pseudomonas aeruginosa* strains: (**A**) Analysis of multi-drug resistance patterns among the strains; (**B**) Expression levels of virulence genes in the studied strains; (**C**) Multi-drug resistance spectrum displaying the distribution of resistance across various antimicrobials; and (**D**) Assessment of biofilm-forming ability across the 63 *Pseudomonas aeruginosa* isolates. The columns indicate the number of strains, with the point-fold line.

The results in Figure 2A highlight that three primary types of antibiotic resistance were prevalent among 26 strains of *Pseudomonas aeruginosa*, with most showing resistance to penicillin, streptomycin, and gentamicin, accounting for 41.27% of the total, as detailed in Figure 2C. According to Table S5, strain ST277 exhibited the highest resistance rates across the board, with 23.53% for tetracycline, 81.81% for streptomycin, and 63.64% for gentamicin. In contrast, strain ST463 displayed relatively less resistance, with a 50% resistance rate for streptomycin and gentamicin. Further analysis revealed intermediate resistance to piperacillin among all five ST types, with ST450 showing the highest incidence at 50%. The resistance patterns among the five ST types predominantly featured triple resistance, particularly notable in ST277, which included two strains with resistance to four antimicrobials and one strain resistant to five different antimicrobials. This pattern underscores the complex resistance profiles across different ST types in *Pseudomonas aeruginosa*.

### 4.2. Correlation Between Virulence Gene Detection and ST Type of 63 Pseudomonas aeruginosa Strains

Table S6 shows that fifteen virulence genes in the 63 *Pseudomonas aeruginosa* strains were detected, of which exoT, exoY, and plcH were detected at a rate of 100%, followed by lasI and rhlR, which were detected at a rate of 93.65%. However, exoS, algD, aprA, and lasB were only detected in one strain. In comparison, the lasA gene had the lowest detection rate of 28.57%, with 45 strains not detected. The detection rates of the remaining five virulence genes (pyo, toxA, lasR, rhlL, and exoU) ranged from 65% to 81%. As shown in Figure 2B, among the strains examined, only five harbored ten or fewer virulence genes: three strains exhibited seven, eight, and nine genes, respectively, while two strains each possessed ten genes. In addition, 92.06% of the strains (58 strains) carried more than 11 virulence genes, with the highest number of strains carrying 14 virulence genes (19 strains, accounting for 30.16% of the total), followed by strains carrying 13 virulence genes (26.98%). Notably, seven strains of *Pseudomonas aeruginosa* were detected in all 15 virulence genes.

For the 63 *Pseudomonas aeruginosa* strains, we focused our statistical analysis on six virulence genes—exoU, pyo, toxA, lasA, lasR, and rhlL—which were selected due to their significant variability in detection across different ST types. This contrasts with other virulence genes, which were ubiquitously present, making these six genes particularly informative for dissecting the strain-specific pathogenic mechanisms and resistance patterns. Table 2 shows that there were significant differences in the detection of virulence genes including exoU, toxA, and lasR in the five major ST types (*p* < 0.05). In ST450, ST571, and ST641, the exoU gene was present in at least five strains per type, with ST571 showing universal presence, as evidenced by a 100% detection rate. toxA was detected in ST571, ST641, and ST463, but not in the ST277 strain, and the significant difference was extremely obvious. The detection rate of lasR in ST450, ST571, and ST463 was at least 75%, among which ST571 was the most detected. In ST571, the detection rate of these three virulence genes (exoU, toxA, and lasR) was 100%.

### 4.3. Correlation of Biofilm Formation and Drug Resistance Phenotypes of 63 Pseudomonas aeruginosa Strains

As shown in Figure 2D, the biofilm-forming ability of the 63 *Pseudomonas aeruginosa* strains was relatively evenly distributed. Strains with moderate biofilm-forming abilities were the most common (27 strains, 42.86%), while fewer strains were characterized by strong biofilm-forming abilities (12 strains, 19.05%). The remaining 38.10% of strains showed a weak or no biofilm-forming ability.

We selected five antimicrobials for the statistical analysis of resistance phenotypes and their relationship with biofilm formability. The results of the Chi-squared test (Table 3) showed that the resistance phenotypes of ciprofloxacin were positively correlated with biofilm formation capacity (χ^2^ = 7.547, *p* = 0.023). Among the ciprofloxacin-susceptible strains, 14.29% (eight strains) of the isolates had a strong biofilm-forming ability, while the number of strains with weak and intermediate biofilm-forming ability was similar and accounted for more than 40%. Among the strains moderately susceptible to ciprofloxacin, the percentage of strains with a strong biofilm-forming ability, weak biofilm-forming ability, and moderate biofilm-forming ability was 57.14% (four strains), 14.29% (one strain), and 28.57% (two strains), respectively. In addition, strains with strong biofilm-forming abilities accounted for more tetracycline, streptomycin, gentamicin, and piperacillin resistance than strains with weak biofilm formabilities.

### 4.4. Correlation of Biofilm Formation and Virulence Genes of 63 Pseudomonas aeruginosa Strains

Six virulence genes were statistically analyzed for their biofilm formation abilities, and the results are shown in Table 4; exoU (χ^2^ = 11.87, *p* = 0.003) and toxA (χ^2^ = 6.267, *p* = 0.044) were negatively correlated with the biofilm formation ability. The strains without exoU and toxA virulence genes showed strong biofilm-forming abilities at a rate of 47.06% (eight strains) and 40% (six strains), respectively. The remainder of the strains showed moderate or weak biofilm-forming abilities. However, the correlation between ST type and biofilm-forming ability was not significant (*p* > 0.05). Among the ST277-type strains, 72.73% (eight strains) did not carry exoU, and none of the strains carried toxA (results shown in Table 5). It is noteworthy that only those strains that did not possess exoU and toxA showed a strong biofilm formation capacity, with percentages of 50% and 36.36%, respectively.

## 5. Discussion

CLSI guidelines for cattle-specific antimicrobials susceptibility testing are available; therefore, the R/S/I values will be re-evaluated using the appropriate veterinary thresholds. In cases where CLSI data are unavailable, alternative thresholds from the The European Centre for Disease Prevention and Control (ECDC) guidelines for cattle were considered. Additionally, another is the potential for sample contamination, defined as the detection of more than two bacterial species in a single sample. We recognize this concern and took strict measures to minimize contamination risk. All milk samples were collected aseptically, with thorough cleaning and disinfection of the udder and teats before sampling, and were promptly stored and transported under controlled conditions to prevent environmental contamination. While the possibility of contamination cannot be entirely excluded, these precautions were intended to reduce this risk significantly.

Bovine mastitis, which seriously affects milk production and quality in dairy cows and hinders the development of the dairy industry, is mainly caused by the pathogenic bacterial infection of the udder [24]. This study revealed an increasing detection rate of *Pseudomonas aeruginosa* in bovine mastitis samples from northern Jiangsu Province, China. A total of 63 isolated strains were identified for 16 ST types, demonstrating significant genetic homogeneity between the isolates. Moreover, the antibiotic resistance patterns of the strains were diverse. Unsurprisingly, all tested strains showed complete resistance to penicillin while being highly susceptible to carbapenems and ceftazidime. This result is consistent with the well-known intrinsic resistance of *Pseudomonas aeruginosa* to penicillin due to its low-permeability outer membrane and the presence of efflux pumps and beta-lactamases that can degrade or expel the antibiotic. Therefore, the observed resistance to penicillin in this study aligns with the expected characteristics of this pathogen and reflects its inherent resistance mechanisms. Additionally, there was a high prevalence of key virulence genes, and an inverse correlation was noted between the number of virulence genes carried and the ability to form biofilms. These findings highlight the complex nature of *Pseudomonas aeruginosa* infections in bovine mastitis and underscore the need for comprehensive control strategies. Further research is needed to understand the implications of these genetic and phenotypic variations on treatment efficacy and disease progression.

Bacterial typing can be used to understand whether there is homology between different strains for more systematic epidemiological analysis [25]. This study identified five dominant ST types of *Pseudomonas aeruginosa*, which together accounted for 65% of the total isolates and were found across various farms. This widespread distribution suggests that these dominant types are potentially transmissible and likely more adaptable to the udder or a common source. Further analysis using an evolutionary tree showed that strains sharing the same ST type tended to cluster on the same branch, indicating a high degree of homology among these isolates. This clustering pattern implies a shared evolutionary pathway, potentially linked to specific environmental or host-associated factors that favor the persistence and spread of these ST types. These findings emphasize the importance of monitoring the spread of dominant ST types and understanding the evolutionary factors that contribute to their prevalence, which can inform targeted control strategies in managing bovine mastitis [26].

The resistance mechanisms of *Pseudomonas aeruginosa* and prolonged antibiotic use have led to widespread resistance [9]. According to a study reported previously, 2722 *Pseudomonas aeruginosa* strains exhibited resistance to imipenem, meropenem, and ceftazidime at rates of 21.9%, 15.4%, and 15.2%, respectively [27]. In this study, distinct antibiotic resistance phenotypes were observed among the five major ST types. For instance, strains resistant to streptomycin were found exclusively in ST641, while resistance to piperacillin was specific to ST277 and ST450. The resistance rates varied significantly across ST types, which is consistent with the findings of the research in the Chinese Burn Center from 2011 to 2016 [28]. Moreover, even within the same ST type, the resistance phenotypes were not uniform, indicating that antibiotic resistance in strains may be driven by their unique genetic adaptations [29]. This phenomenon is consistent with published findings highlighting that *Pseudomonas aeruginosa* isolates belonging to high-risk clones such as ST235 and ST111 exhibited significant variability in resistance phenotypes, driven by diverse resistance mechanisms including efflux pumps and β-lactamases [30].

The pathogenic potential of *Pseudomonas aeruginosa* largely depends on its virulence factors and regulatory genes, including exotoxins, alkaline protease, elastase, pyocyanin, and T3SS effector proteins. In the current study, with the investigation of 63 strains, we discovered that 15 virulence genes were highly prevalent, indicating the significant pathogenicity of the opportunistic bacterium, *Pseudomonas aeruginosa* [31]. Almost all of the isolates carried algD and aprA, suggesting robust adaptability to external environments. Notably, 45 strains contained both ExoS and ExoU—a rare occurrence in *Pseudomonas aeruginosa* [32]—likely due to ExoU’s transfer via genomic islands, which is suggested to be related to environmental adaptability [33]. Moreover, six key virulence genes (exoU, pyo, toxA, lasA, lasR, and rhlL) were less frequent in strains resistant to penicillin, streptomycin, and gentamicin, suggesting an association between antibiotic resistance and virulence profiles [34]. In particular, exoU, toxA, and lasR were prevalent in ST571, whereas toxA was absent in ST277, highlighting a possible correlation between virulence gene distribution and specific ST types. The distribution of virulence genes across different ST types suggests a potential association between genetic lineage and pathogenic potential. Further investigation into the functional implications of these virulence gene profiles could inform the development of more targeted treatment approaches.

Biofilms play a significant role in the persistence of bacterial infections, with 65%-80% of infections linked to biofilm formation [35]. Biofilms can increase bacterial resistance to immune defenses and antimicrobials by 10 to 1000 times, enhancing mortality [36,37]. However, our study observed fewer strong biofilm-forming strains, with weaker biofilm-forming abilities being more prevalent. It is likely that the samples from bovine mastitis may exhibit less robust biofilm capabilities compared to strains from human infections [38]. The reduced biofilm-forming ability of bovine-derived pathogens compared to their human-derived counterparts may stem from several factors, including divergent host environments, immune responses, and pathogen–host interactions. Khoramian et al. (2015) demonstrated that *Staphylococcus aureus* strains originating from human infections exhibited a stronger biofilm-forming capacity than those from bovine sources, a difference attributed to the distinct survival challenges faced within their respective hosts [39]. Similarly, Horiuk et al. (2019) found that *Staphylococcus aureus*, a primary pathogen in bovine mastitis, showed significantly lower biofilm-forming capabilities compared to certain human pathogens, further underscoring the role of environmental and biological factors in influencing biofilm development [40]. Our study identified a significant inverse relationship between biofilm formation capacity and the presence of certain virulence genes, such as exoU and toxA. This finding suggests a potential trade-off in *Pseudomonas aeruginosa* between biofilm formation and acute virulence. The underlying mechanisms of this trade-off are likely complex and multifactorial, involving intricate regulatory pathways that balance survival and persistence through biofilm formation against acute virulence aimed at exploiting host vulnerabilities [41]. One possible explanation for this phenomenon is that biofilm formation serves as a survival strategy in environments where long-term persistence is advantageous, such as within the udder of a dairy cow [42]. In such settings, biofilm formation may provide a significant advantage by shielding bacterial communities from host defenses and antibiotic treatments. Conversely, the downregulation of acute virulence factors such as exoU and toxA might attenuate the immediate immune response, allowing the bacteria to remain undetected for extended periods.

The T3SS mechanism is critical for cytotoxicity and invasive activities, allowing *Pseudomonas aeruginosa* to inject virulence proteins directly into host cells, facilitating infection [43]. We detected four T3SS-secreted virulence genes at high levels, with exoU being notably potent and toxA highly lethal. However, strong biofilm-forming strains exhibited lower levels of exoU and toxA, suggesting an inverse relationship between biofilm formation and virulence gene carriage. This pattern indicates that strains with greater biofilm-forming capacities might downregulate the expression of certain virulence factors to prioritize biofilm development, potentially reducing their overall virulence [44]. This trade-off suggests that *Pseudomonas aeruginosa* may prioritize either acute virulence or persistence, depending on the host environment and selective pressures. Such adaptive plasticity likely contributes to the bacterium’s success as a mastitis pathogen and poses significant challenges for developing effective control measures [45]. The inverse relationship between biofilm formation and certain virulence factors indicates a complex adaptive strategy employed by *Pseudomonas aeruginosa* in bovine mastitis. Future research should aim to elucidate the regulatory mechanisms that govern this balance, as they may reveal novel targets for therapeutic intervention.

This study elucidates the high genetic homogeneity, varied antibiotic resistance, and complex interplay between the virulence factors and biofilm-forming abilities of *Pseudomonas aeruginosa* isolated from bovine mastitis in northern Jiangsu Province. These insights enhance our understanding of the pathogen’s behavior in the context of bovine mastitis and underscore the significant challenges in developing effective control strategies. Future research should focus on deciphering the molecular mechanisms driving the observed phenotypic variations and exploring innovative approaches to disrupt the pathogen’s adaptive strategies. This knowledge will be pivotal in developing more targeted and effective interventions to mitigate the impact of *Pseudomonas aeruginosa* on animal health and the dairy industry.

## 6. Conclusions

This study identified 63 strains of *Pseudomonas aeruginosa* from bovine mastitis samples collected in northern Jiangsu Province, which were classified into 16 ST types. Five predominant ST types accounted for 65% of the isolates, indicating a notable degree of genetic homogeneity. The strains exhibited complete resistance to penicillin and high levels of resistance to streptomycin, while remaining highly susceptible to carbapenems and ceftazidime. An inverse correlation was observed between the number of virulence genes and biofilm formation capacity, with strains demonstrating strong biofilm-forming abilities and showing enhanced antibiotic resistance. These findings highlight the need for targeted prevention and therapeutic strategies to effectively manage *Pseudomonas aeruginosa* in bovine mastitis.

## Figures and Tables

**Table 1:** Results of antimicrobial susceptibility test of 63 *Pseudomonas aeruginosa* strains.

	Resistance Number	Resistance Rate	Intermediate Number	Intermediate Rate	Sensitive Number	Sensitive Rate	R/I/S Breakpoint (mg/mL)
Tetracycline	15	23.81%	1	1.59%	47	74.60%	≥16/8/≤4
Streptomycin	44	69.84%	3	4.76%	16	25.40%	≥16/8/≤4
Ciprofloxacin	0	0.00%	7	11.11%	56	88.89%	≥2/1/≤0.5
Gentamicin	31	49.21%	7	11.11%	25	39.68%	≥16/8/≤4
Imipenem	0	0.00%	1	1.59%	62	98.41%	≥8/4/≤2
Meropenem	0	0.00%	0	0.00%	63	100.00%	≥8/4/≤2
Piperacilln	5	7.94%	13	20.63%	45	71.43%	≥64/32/≤16
Ceftazidime	0	0.00%	0	0.00%	63	100.00%	≥32/16/≤8
Penicillin	63	100.00%	0	0.00%	0	0.00%	≥64/32/≤16
Cefotaxime	1	1.59%	0	0.00%	62	98.41%	≥32/16/≤8

**Table 2:** The correlation between five main ST types and virulence genes in 63 isolates of *Pseudomonas aeruginosa*.

Virulence Gene	ST277	ST450	ST571	ST641	ST463	*p* Value
exoU	3 (27.27%)	7 (87.5%)	8 (100%)	5 (62.5%)	3 (50%)	0.010
pyo	8 (72.73%)	8 (100%)	7 (87.5%)	7 (87.5%)	3 (50%)	0.170
toxA	0 (0%)	7 (87.5%)	8 (100%)	8 (100%)	6 (100%)	0.001
lasA	9 (81.82%)	1 (12.5%)	4 (50%)	3 (37.5%)	3 (50%)	0.051
lasR	6 (54.55%)	6 (75%)	8 (100%)	2 (25%)	5 (83.33%)	0.019
rhlL	9 (81.82%)	6 (75%)	7 (87.5%)	6 (75%)	5 (83.33%)	0.963

**Table 3:** Relationship between antibiotic resistance and biofilm formation in 63 isolates of *Pseudomonas aeruginosa*.

Antibiotic		Biofilm Forming Ability			χ^2^	*p*
		Weak	Medium	Strong		
Tetracycline	R	5	6	4	2.338	0.674
	S	18	21	8		
	I	1	0	0		
Streptomycin	R	15	19	10	2.2548	0.636
	S	8	6	2		
	I	1	2	0		
Ciprofloxacin	R	0	0	0	7.547	0.023
	S	23	25	8		
	I	1	2	4		
Gentamicin	R	10	12	9	4.861	0.302
	S	12	11	2		
	I	2	4	1		
Piperacilln	R	1	1	3	6.646	0.156
	S	17	20	8		
	I	6	6	1		

**Table 4:** The relationship between the detection of virulence genes and the ability of biofilm formation in 63 isolates of *Pseudomonas aeruginosa*.

Virulence Genes		Biofilm Forming Ability			χ^2^	*p*
		Weak	Medium	Strong		
exoU	+	20	22	4	11.87	0.003
	-	4	5	8		
pyo	+	20	21	10	0.309	0.857
	-	4	6	2		
toxA	+	21	21	6	6.267	0.044
	-	3	6	6		
lasA	+	4	8	6	4.381	0.112
	-	20	19	6		
lasR	+	18	17	6	2.293	0.318
	-	6	10	6		
rhlL	+	20	21	9	0.412	0.814
	-	4	6	3		

**Table 5:** Relationships between ST type and exoU/toxA and biofilm formation in 63 isolates of *Pseudomonas aeruginosa*.

ST	Virulence Gene	Biofilm Forming Capacity			Total
	exoU/toxA	Strong	Medium	Weak	
277	+	0/0	2/0	1/0	3/0
	-	4/4	2/4	2/3	8/11
450	+	0/0	3/2	4/5	7/7
	-	0/0	0/1	1/0	1/1
571	+	1/1	1/1	6/6	8/8
	-	0/0	0/0	0/0	0/0
641	+	0/2	3/4	2/2	5/8
	-	2/0	1/0	0/0	3/0
463	+	0/1	1/2	2/3	3/6
	-	1/0	1/0	1/0	3/0

## Data Availability

The data presented in this study are available on request from the corresponding author.

## Supplementary Materials

The following supporting information can be downloaded at: https://www.mdpi.com/article/10.3390/ani14223290/s1, Table S1: 16S rDNA primer sequence information; Table S2: Gene and primer sequence information of *Pseudomonas aeruginosa*; Table S3: Isolation of pathogenic bacteria from clinical mastitis samples of dairy cows; Table S4: ST-type and number of strains of *Pseudomonas aeruginosa*; Table S5: Drug resistance of five main ST types; Table S6: Detection of virulence gene in 63 strains of *Pseudomonas aeruginosa*.

## References

[1] Heikkilä A.-M., Liski E., Pyörälä S., Taponen S. (2018). Pathogen-specific production losses in bovine mastitis. J. Dairy Sci..

[2] Čobirka M., Tančin V., Slama P. (2020). Epidemiology and classification of mastitis. Animal.

[3] Gonçalves J.L., Kamphuis C., Martins C., Barreiro J.R., Tomazi T., Gameiro A.H., Hogeveen H., dos Santos M.V. (2018). Bovine subclinical mastitis reduces milk yield and economic return. Livest Sci..

[4] Amoh T., Murakami K., Kariyama R., Hori K., Viducic D., Hirota K., Igarashi J., Suga H., Parsek M.R., Kumon H. (2017). Effects of an autoinducer analogue on antibiotic tolerance in *Pseudomonas aeruginosa*. J. Antimicrob. Chemother..

[5] Li X., Ye Y., Zhou X., Huang C., Wu M. (2015). Atg7 enhances host defense against infection via down-regulation of superoxide but up-regulation of nitric oxide. J. Immunol..

[6] Demirdjian S., Sanchez H., Hopkins D., Berwin B. (2019). Motility-Independent Formation of Antibiotic-Tolerant *Pseudomonas aeruginosa* Aggregates. Appl. Environ. Microbiol..

[7] Pérez-Losada M., Cabezas P., Castro-Nallar E., Crandall K.A. (2013). Pathogen typing in the genomics era: MLST and the future of molecular epidemiology. Infect. Genet. Evol..

[8] Jolley K.A., Bray J.E., Maiden M.C.J. (2018). Open-access bacterial population genomics: BIGSdb software, the PubMLST.org website and their applications. Wellcome Open Res..

[9] Azab K.S.M., Abdel-Rahman M.A., El-Sheikh H.H., Azab E., Gobouri A.A., Farag M.M.S. (2021). Distribution of Extended-Spectrum β-Lactamase (ESBL)-Encoding Genes among Multidrug-Resistant Gram-Negative Pathogens Collected from Three Different Countries. Antibiotics.

[10] Wolfmeier H., Wardell S.J.T., Liu L.T., Falsafi R., Draeger A., Babiychuk E.B., Pletzer D., Hancock R.E.W. (2022). Targeting the *Pseudomonas aeruginosa* Virulence Factor Phospholipase C With Engineered Liposomes. Front. Microbiol..

[11] Bleves S., Viarre V., Salacha R., Michel G.P., Filloux A., Voulhoux R. (2010). Protein secretion systems in *Pseudomonas aeruginosa*: A wealth of pathogenic weapons. Int. J. Med. Microbiol..

[12] Gervasoni L.F., Peixoto I.C., Imperador A.C., De Oliveira L.B., Correia L.F., Vieira K.C.d.O., Saeki E.K., Lima P.E.d.S., Mareco E.A., Pereira V.C. (2023). Relationship between antibiotic resistance, biofilm formation, virulence factors and source of origin of *Pseudomonas aeruginosa* environmental isolates with regard to the presence of metallo-β-lactamase-encoding genes. Microb. Pathog..

[13] Zhang Y., Zhang C., Du X., Zhou Y., Kong W., Lau G.W., Chen G., Kohli G.S., Yang L., Wang T. (2019). Glutathione Activates Type III Secretion System Through Vfr in *Pseudomonas aeruginosa*. Front. Cell Infect. Microbiol..

[14] Guo M., Feng C., Ren J., Zhuang X., Zhang Y., Zhu Y., Dong K., He P., Guo X., Qin J. (2017). A Novel Antimicrobial Endolysin, LysPA26, against *Pseudomonas aeruginosa*. Front. Microbiol..

[15] Ghanbari A., Dehghany J., Schwebs T., Müsken M., Häussler S., Meyer-Hermann M. (2016). Inoculation density and nutrient level determine the formation of mushroom-shaped structures in *Pseudomonas aeruginosa* biofilms. Sci. Rep..

[16] Thi M.T.T., Wibowo D., Rehm B.H.A. (2020). *Pseudomonas aeruginosa* Biofilms. Int. J. Mol. Sci..

[17] Kumar S., Stecher G., Tamura K. (2016). MEGA7: Molecular Evolutionary Genetics Analysis Version 7.0 for Bigger Datasets. Mol. Biol. Evol..

[18] Curran B., Jonas D., Grundmann H., Pitt T., Dowson C.G. (2004). Development of a multilocus sequence typing scheme for the opportunistic pathogen *Pseudomonas aeruginosa*. J. Clin. Microbiol..

[19] Spinler J.K., Raza S., Thapa S., Venkatachalam A., Scott T., Runge J.K., Dunn J., Versalovic J., Luna R.A. (2022). Comparison of Whole Genome Sequencing and Repetitive Element PCR for Multidrug-Resistant *Pseudomonas aeruginosa* Strain Typing. J. Mol. Diagn..

[20] Clinical and Laboratory Standards Institute (CLSI) (2020). Performance Standards for Antimicrobial Disk and Dilution Susceptibility Tests for Bacteria Isolated from Animals.

[21] Zhang Y., Chen R., Wang Y., Wang P., Pu J., Xu X., Chen F., Jiang L., Jiang Q., Yan F. (2022). Antibiofilm activity of ultra-small gold nanoclusters against *Fusobacterium nucleatum* in dental plaque biofilms. J. Nanobiotechnology.

[22] Ou X., Yang B., Chen J. (2021). Analysis of film-forming capacity of biofilms in vitro models of common bacterial biofilms. Orient. Medicat. Diet..

[23] (2017). General Requirements for the Competence of Testing and Calibration Laboratories.

[24] Hernandez L., Bottini E., Cadona J., Cacciato C., Monteavaro C., Bustamante A., Sanso A.M. (2021). Multidrug Resistance and Molecular Characterization of Streptococcus agalactiae Isolates From Dairy Cattle With Mastitis. Front. Cell Infect. Microbiol..

[25] Schürch A.C., Arredondo-Alonso S., Willems R.J.L., Goering R.V. (2018). Whole genome sequencing options for bacterial strain typing and epidemiologic analysis based on single nucleotide polymorphism versus gene-by-gene-based approaches. Clin. Microbiol. Infect..

[26] Mayer K., Kucklick M., Marbach H., Ehling-Schulz M., Engelmann S., Grunert T. (2021). Within-Host Adaptation of *Staphylococcus aureus* in a Bovine Mastitis Infection Is Associated with Increased Cytotoxicity. Int. J. Mol. Sci..

[27] Hancock R.E. (1998). Resistance mechanisms in *Pseudomonas aeruginosa* and other nonfermentative gram-negative bacteria. Clin. Infect. Dis..

[28] Yin S., Chen P., You B., Zhang Y., Jiang B., Huang G., Yang Z., Chen Y., Chen J., Yuan Z. (2018). Molecular Typing and Carbapenem Resistance Mechanisms of *Pseudomonas aeruginosa* Isolated From a Chinese Burn Center from 2011 to 2016. Front. Microbiol..

[29] Smith E.E., Buckley D.G., Wu Z., Saenphimmachak C., Hoffman L.R., D’argenio D.A., Ramsey B.W., Speert D.P., Moskowitz S.M., Burns J.L. (2006). Genetic adaptation by *Pseudomonas aeruginosa* to the airways of cystic fibrosis patients. Proc. Natl. Acad. Sci. USA.

[30] Almaghrabi R.S., Macori G., Sheridan F., McCarthy S.C., Floss-Jones A., Fanning S., Althawadi S., Mutabagani M., Binsaslloum A., Alrasheed M. (2024). Whole genome sequencing of resistance and virulence genes in multi-drug resistant *Pseudomonas aeruginosa*. J. Infect. Public Health.

[31] She P., Liu Y., Luo Z., Chen L., Zhou L., Hussain Z., Wu Y. (2020). PA2146 Gene Knockout Is Associated With *Pseudomonas aeruginosa* Pathogenicity in Macrophage and Host Immune Response. Front. Cell Infect. Microbiol..

[32] Díaz-Ríos C., Hernández M., Abad D., Álvarez-Montes L., Varsaki A., Iturbe D., Calvo J., Ocampo-Sosa A.A. (2021). New Sequence Type ST3449 in Multidrug-Resistant *Pseudomonas aeruginosa* Isolates from a Cystic Fibrosis Patient. Antibiotics.

[33] Wolfgang M.C., Kulasekara B.R., Liang X., Boyd D., Wu K., Yang Q., Miyada C.G., Lory S. (2003). Conservation of genome content and virulence determinants among clinical and environmental isolates of *Pseudomonas aeruginosa*. Proc. Natl. Acad. Sci. USA.

[34] Subedi D., Vijay A.K., Kohli G.S., Rice S.A., Willcox M. (2018). Association between possession of ExoU and antibiotic resistance in *Pseudomonas aeruginosa*. PLoS ONE.

[35] Rahman M.A., Amirkhani A., Chowdhury D., Mempin M., Molloy M.P., Deva A.K., Vickery K., Hu H. (2022). Proteome of *Staphylococcus aureus* Biofilm Changes Significantly with Aging. Int. J. Mol. Sci..

[36] Sharma D., Misba L., Khan A.U. (2019). Antibiotics versus biofilm: An emerging battleground in microbial communities. Antimicrob. Resist. Infect. Control.

[37] Dadgostar P. (2019). Antimicrobial Resistance: Implications and Costs. Infect. Drug Resist..

[38] Radwan E.-Z., Ebshahy E.M., Khalil S.A., Torky H.A. (2021). Relation Between Biofilm Formation and Resistance to Antibacterial Agents of *Pseudomonas aeruginosa* Isolated from Different Sources. Alex. J. Vet. Sci..

[39] Khoramian B., Jabalameli F., Niasari-Naslaji A., Taherikalani M., Emaneini M. (2015). Comparison of virulence factors and biofilm formation among *Staphylococcus aureus* strains isolated from human and bovine infections. Microb. Pathog..

[40] Horiuk Y., Kukhtyn M., Kovalenko V., Kornienko L., Horiuk V., Liniichuk N. (2019). Biofilm formation in bovine mastitis pathogens and the effect on them of antimicrobial drugs. Indep. J. Manag. Prod..

[41] Ferenci T. (2016). Trade-off Mechanisms Shaping the Diversity of Bacteria. Trends Microbiol..

[42] Gomes F., Saavedra M.J., Henriques M. (2016). Bovine mastitis disease/pathogenicity: Evidence of the potential role of microbial biofilms. Pathog. Dis..

[43] Lin Q., Pilewski J.M., Di Y.P. (2021). Acidic Microenvironment Determines Antibiotic Susceptibility and Biofilm Formation of *Pseudomonas aeruginosa*. Front. Microbiol..

[44] Huang P., Li Z., Liu R., Bartlam M., Wang Y. (2024). Polystyrene nanoparticles induce biofilm formation in *Pseudomonas aeruginosa*. J. Hazard. Mater..

[45] Stepanyan K., Wenseleers T., Duéñez-Guzmán E.A., Muratori F., Van den Bergh B., Verstraeten N., De Meester L., Verstrepen K.J., Fauvart M., Michiels J. (2015). Fitness trade-offs explain low levels of persister cells in the opportunistic pathogen *Pseudomonas aeruginosa*. Mol. Ecol..

