# Supplementary material for: Characterization of Pseudomonas aeruginosa Isolated from Bovine Mastitis in Northern Jiangsu Province and Correlation to Drug Resistance and Biofilm Formability
Source: Animals (Basel). 2024 Nov 15;14(22):3290. doi: 10.3390/ani14223290 (PMC11590879; doi:10.3390/ani14223290)
Supplement: Supplementary file 1 [file animals-14-03290-s001.zip › animals-3210843-supplementary.pdf]

**Table S1** 16S rDNA primer sequence information

| Name | Sequence | bp Length |
|---|---|---|
| 16S rDNA 27 F | 5′ -AGAGTTTGATCCTGGCTCAG-3′ | 1 500 |
| 16S rDNA 1492 R | 5′ -TACGGCTACCTTGTTACGACT-3′ | |

**Table S2** Gene and primer sequence information of *Pseudomonas aeruginosa*

| Gene | primer | Sequence(5´-3´) | bp Length |
|---|---|---|---|
| acsA | acsA-F | ACCTGGTGTACGCCTCGCTGAC | 842 |
| | acsA-R | GACATAGATGCCCTGCCCCTTGAT | |
| aroE | aroE-F | TGGGGCTATGACTGGAAACC | 1056 |
| | aroE-R | TAACCCGGTTTTGTGATTCCTACA | |
| guaA | guaA-F | CGGCCTCGACGTGTGGATGA | 940 |
| | guaA-R | GAACGCCTGGCTGGTCTTGTGGTA | |
| mutL | mutL-F | CCAGATCGCCGCCGGTGAGGTG | 487 |
| | mutL-R | CAGGGTGCCATAGAGGAAGTC | |
| nuoD | nuoD-F | ACCGCCACCCGTACTG | 1024 |
| | nuoD-R | TCTCGCCCATCTTGACCA | |
| ppsA | ppsA-F | GGTCGCTCGGTCAAGGTAGTGG | 989 |
| | ppsA-R | GGGTTCTCTTCTTCCGGCTCGTAG | |
| trpE | trpE-F | GCGGCCCAGGGTCGTGAG | 811 |
| | trpE-R | CCCGGCGCTTGTTGATGGTT | |
| exoS | exoS_F | TCAGCAGAGTCCGTCTTTCGCC | 407 |
| | exoS_R | GCCAGGCGGGAGTGCTCCCGG | |
| exoT | exoT_F | TCAGCAGAACCCGTCTTTCGT | 407 |
| | exoT_R | GCCAGGCGCGTGTGATCCTTC | |
| exoU | exoU_F | CCGTCGCAGGCAGCGCATAAGTCC | 420 |
| | exoU_R | GAACGCCGCCGGGCTCATACCTGA | |
| exoY | exoY_F | ACCATGCGTATCGACGGTCATC | 323 |
| | exoY_R | TTGCTGAGATGCTGGTCGACAC | |
| Pyo | Pyo_F | TGCCGGTACGACTCACGAGTG | 231 |

| | | | |
|---|---|---|---|
| | Pyo_R | GTTCTGGCTTCCTGGAGGGGT | |
| aprA | aprA_F | CAGACCCTGACCCACGAGAT | 452 |
| | aprA_R | CATTGCCCTTCAACCCG | |
| toxA | toxA_F | GGTAACCAGCTCAGCCACA | 301 |
| | toxA_R | TGCCTTCCCAGGTATCGT | |
| lasA | lasA_F | GCAGCACAAAAGATCCC | 1075 |
| | lasA_R | GAAATGCAGGTGCGGTC | |
| lasB | lasB_F | CGTCTCCTACCTGATTCCCG | 413 |
| | lasB_R | GCACCTTCATGTACAGCTTGTG | |
| plcH | plcH_F | GCACGTGGTCATCCTGATGC | 608 |
| | plcH_R | TCCGTAGGCGTCGACGTAC | |
| algD | algD_F | ATCAGCATCTTTGGTTTGGG | 346 |
| | algD_R | TGTGGCGTTCGGACTTCT | |
| lasI | lasI_F | CGTGCTCAAGTGTTCAAGG | 295 |
| | lasI_R | TACAGTCGGAAAAGCCCAG | |
| lasR | lasR_F | AAGTGGAAAATTGGAGTGGAG | 130 |
| | lasR_R | GTAGTTGCCGACGACGATGAAG | |
| rhlL | rhlL_F | TTCATCCTCCTTTAGTCTTCCC | 155 |
| | rhlL_R | TTCCAGCGATTCAGAGAGC | |
| rhlR | rhlR_F | TGCATTTATCGATCAGGGC | 133 |
| | rhlR_R | CACTTCCTTTTCCAGGACG | |

**Table S3** Isolation of pathogenic bacteria from clinical mastitis samples of dairy cows

| Bacteria | Number | Rate |
|---|---|---|
| *Escherichia coli* | 65 | 15.33% |
| *Klebsiella Pneumoniae* | 58 | 13.68% |
| *Pseudomonas aeruginosa* | 63 | 14.86% |
| *Staphylococcus spp.* | 97 | 22.88% |
| *Streptococcus spp.* | 18 | 4.25% |
| *Enterococcus Thiercelin and Jouhaud spp.* | 15 | 3.54% |
| *Bacillus Cohn spp.* | 61 | 14.39% |
| Other | 47 | 11.08% |
| Total | 424 | 100% |

**Table S4** ST-type and number of strains of *Pseudomonas aeruginosa*

| ST | acsA | aroE | guaA | mutL | nuoD | ppsA | trpE | strains |
|---|---|---|---|---|---|---|---|---|
| 277 | 39 | 5 | 9 | 11 | 27 | 5 | 85 | 11 |
| 450 | 7 | 5 | 83 | 77 | 3 | 4 | 34 | 8 |
| 571 | 198 | 20 | 26 | 9 | 3 | 64 | 69 | 8 |
| 641 | 6 | 5 | 6 | 5 | 4 | 4 | 81 | 8 |
| 463 | 6 | 5 | 5 | 43 | 91 | 6 | 83 | 6 |
| 185 | 6 | 5 | 5 | 31 | 91 | 6 | 83 | 5 |
| 836 | 6 | 5 | 1 | 5 | 27 | 76 | 81 | 3 |
| 1061 | 92 | 75 | 26 | 31 | 4 | 120 | 46 | 3 |
| 510 | 104 | 75 | 26 | 71 | 4 | 4 | 157 | 3 |
| 147 | 185 | 5 | 6 | 11 | 3 | 87 | 87 | 2 |
| 256 | 6 | 5 | 5 | 54 | 3 | 200 | 96 | 1 |
| 594 | 198 | 5 | 146 | 31 | 14 | 4 | 81 | 1 |
| 640 | 25 | 20 | 212 | 5 | 3 | 76 | 85 | 1 |
| 752 | 198 | 5 | 146 | 43 | 14 | 87 | 81 | 1 |
| 838 | 6 | 5 | 1 | 18 | 3 | 87 | 83 | 1 |
| 2197 | 91 | 75 | 3 | 43 | 13 | 162 | 70 | 1 |

**Table S5** Drug resistance of five main ST types

| ST | Tetracycline | | Streptomycin | | Ciprofloxacin | | Gentamicin | | Piperacilln | |
|---|---|---|---|---|---|---|---|---|---|---|
| | Resistance | Intermediate | Resistance | Intermediate | Resistance | Intermediate | Resistance | Intermediate | Resistance | Intermediate |
| ST277 | 23.53%（4/11） | 0 | 81.81%（9/11） | 0 | 0 | 9.15%（1/11） | 63.64%（7/11） | 18.19% (2/11) | 9.15%（1/11） | 18.19% (2/11) |
| ST450 | 12.50%（1/8） | 0 | 50%（4/8） | 0 | 0 | 0 | 37.50%（3/8） | 0 | 12.50%（1/8） | 37.50%（3/8） |
| ST571 | 12.50%（1/8） | 0 | 75%（6/8） | 0 | 0 | 12.50%（1/8） | 50%（4/8） | 0 | 0 | 25%（2/8） |
| ST641 | 12.50%（1/8） | 12.50%（1/8） | 62.5%（5/8） | 37.50%（3/8） | 0 | 25%（2/8） | 50%（4/8） | 12.50%（1/8） | 0 | 12.50%（1/8） |
| ST463 | 0 | 0 | 50%（3/6） | 0 | 0 | 16.67%（1/6） | 50%（3/6） | 0 | 0 | 16.67%（1/6） |

**Table S6** Detection of virulence gene in 63 strains of *Pseudomonas aeruginosa*

| Virulence gene | Not detected strains | Detected strains | Detected rate |
|---|---|---|---|
| exoS | 1 | 62 | 98.41% |
| exoT | 0 | 63 | 100.00% |
| exoU | 17 | 46 | 73.02% |
| exoY | 0 | 63 | 100.00% |
| pyo | 12 | 51 | 80.95% |
| toxA | 15 | 48 | 76.19% |
| algD | 1 | 62 | 98.41% |
| aprA | 1 | 62 | 98.41% |
| lasA | 45 | 18 | 28.57% |
| lasB | 1 | 62 | 98.41% |
| plcH | 0 | 63 | 100.00% |
| lasI | 4 | 59 | 93.65% |
| lasR | 22 | 41 | 65.08% |
| rhlL | 13 | 50 | 79.37% |
| rhlR | 4 | 59 | 93.65% |
